# Baseline monocyte GIP receptor mRNA expression is associated with reductions in body weight and FIB-4 index after switching to tirzepatide in patients with type 2 diabetes

**DOI:** 10.1007/s13340-026-00940-3

**Published:** 2026-10-31

**Authors:** Hideyuki Okuma

**Affiliations:** Department of Diabetes and Endocrinology, Nerima Hikarigaoka Hospital, 2-5-1 Hikarigaoka, Nerima-ku, Tokyo, 1790072 Japan

**Keywords:** Tirzepatide, GIP receptor, Type 2 diabetes, Monocytes, Fibrosis-4 index

## Abstract

**Background:**

Tirzepatide improves body weight and glycemic control in patients with type 2 diabetes, but factors associated with inter-individual differences after switching remain unclear. We examined whether baseline monocyte glucose-dependent insulinotropic polypeptide receptor (GIPR) mRNA expression was associated with changes in body weight and the fibrosis-4 (FIB-4) index after switching from glucagon-like peptide−1 receptor agonists (GLP-1RAs) to tirzepatide.

**Methods:**

This single-center retrospective study included 90 patients with type 2 diabetes who had received a GLP-1RA for at least 6 months before switching. Baseline monocyte *GIPR* mRNA expression was measured by quantitative real-time polymerase chain reaction. Associations with percentage body-weight change and FIB-4 index change during the 6 months after tirzepatide initiation were assessed by correlation and multivariable regression. Exploratory ex vivo assays were performed to assess monocyte responses to native GIP.

**Results:**

Higher baseline monocyte *GIPR* mRNA expression was associated with greater reductions in body weight and the FIB-4 index and remained significant in multivariable models. The association with the FIB-4 index remained significant after additional adjustment for percentage body-weight change at 6 months after tirzepatide initiation. *GIPR* mRNA expression was not associated with body-weight change during the preceding 6 months of GLP-1RA therapy. Native GIP suppressed lipopolysaccharide-induced interleukin-6 and tumor necrosis factor-alpha responses in monocytes.

**Conclusions:**

Baseline monocyte *GIPR* mRNA expression was associated with body-weight and FIB-4 index reductions after switching to tirzepatide. It may represent an exploratory marker of clinical changes, but treatment specificity, causal GIPR-dependent mechanisms, and structural liver fibrosis regression were not established.

**Graphical abstract:**

**Figure:**
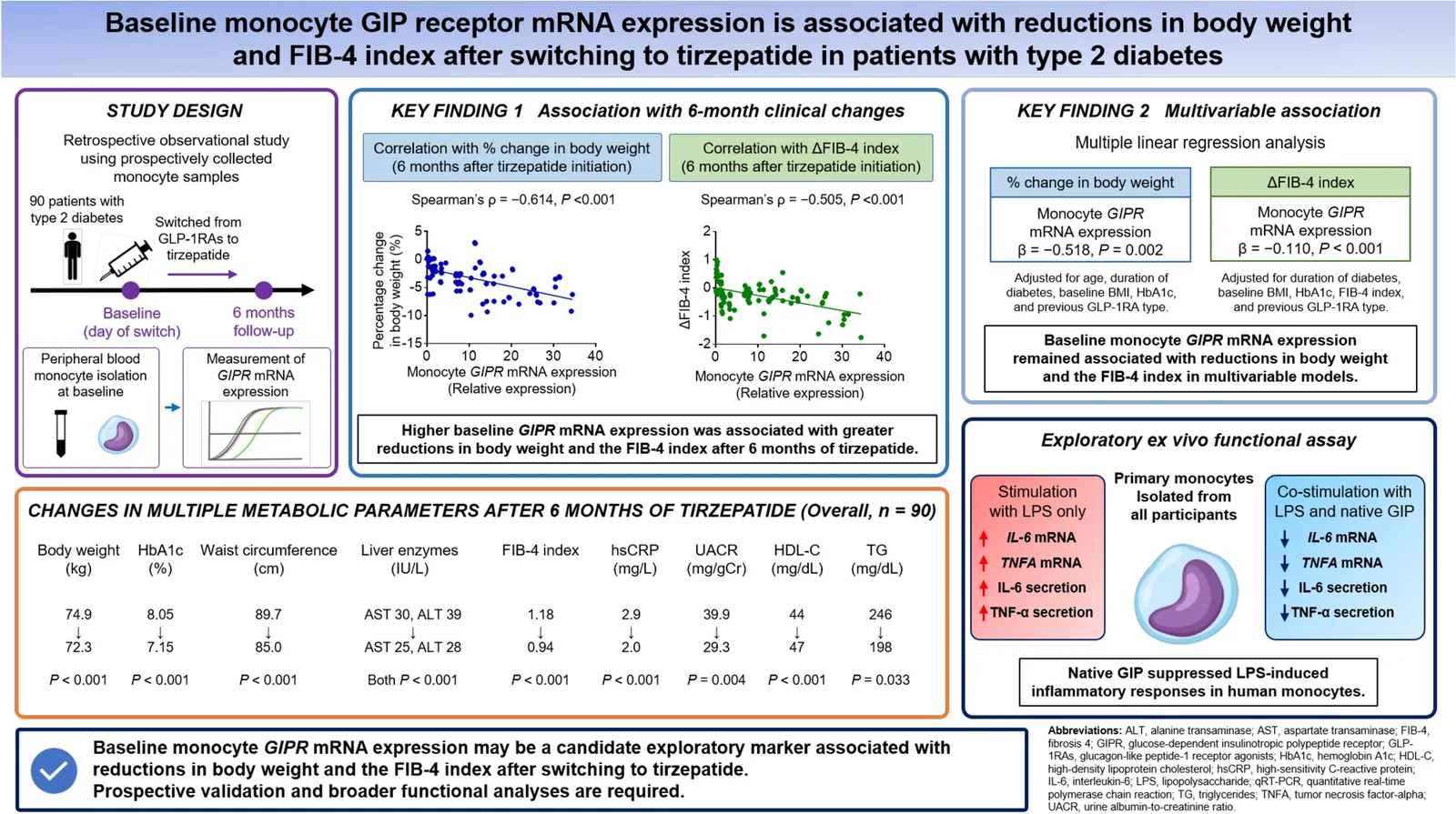

Summary of the study design and main finding. Baseline *GIPR* mRNA expression in peripheral blood monocytes was associated with reductions in body weight and the FIB-4 index after switching to tirzepatide in patients with type 2 diabetes. In exploratory ex vivo assays, native GIP suppressed LPS-induced inflammatory responses, supporting monocyte *GIPR* expression as a candidate exploratory marker requiring further validation.

**Supplementary Information:**

The online version contains supplementary material available at https://doi.org/10.1007/s13340-026-00940-3.

## Introduction

Tirzepatide, a dual glucose-dependent insulinotropic polypeptide (GIP) and glucagon-like peptide−1 (GLP-1) receptor agonist, has shown greater efficacy in reducing body weight and improving glycemic control than conventional GLP-1 receptor agonists (GLP-1RAs) [1, 2]. Recent evidence has also indicated that switching from GLP-1RAs to tirzepatide provides additional metabolic benefits. A prospective study reported further reductions in hemoglobin A1c (HbA1c) and body weight after switching to tirzepatide [3], while the SURPASS-SWITCH trial showed that switching from GLP-1RAs to tirzepatide was superior to dose escalation of GLP-1RAs for improving glycemic control and reducing body weight [4]. In addition, our previous study demonstrated that this switching strategy was associated with favorable changes in metabolic parameters, metabolic dysfunction-associated steatotic liver disease (MASLD)-related non-invasive indices, and inflammation-related markers in patients with type 2 diabetes [5].

Despite these clinical benefits, factors associated with inter-individual variation in clinical changes after switching to tirzepatide remain incompletely understood. Although activation of GIP receptor (GIPR) signaling is a pharmacological component of tirzepatide, the relationship between cell-specific GIPR expression and clinical changes after switching to tirzepatide has not been well characterized. Previous studies have shown that GIPR is expressed in bone marrow-derived myeloid cells, including monocytes and subsets of macrophages [6–8]. GIPR expression appears to be higher in monocytes than in differentiated macrophages [6], suggesting context-dependent regulation during myeloid differentiation. Peripheral blood monocytes therefore represent an accessible cell population in which inter-individual differences in GIPR expression can be examined.

Therefore, this study aimed to investigate whether baseline *GIPR* mRNA expression in peripheral blood monocytes is associated with subsequent clinical changes after switching from GLP-1RAs to tirzepatide in patients with type 2 diabetes. We hypothesized that baseline monocyte *GIPR* mRNA expression, quantified by quantitative real-time polymerase chain reaction (qRT-PCR), may represent a candidate exploratory marker associated with reductions in body weight and the FIB-4 index after switching to tirzepatide. The primary outcomes were percentage change in body weight and change in the fibrosis-4 (FIB-4) index, a routinely available non-invasive fibrosis-related index used for liver fibrosis risk stratification in MASLD.

## Materials and methods

### Study design and participants

**Fig. 1:**
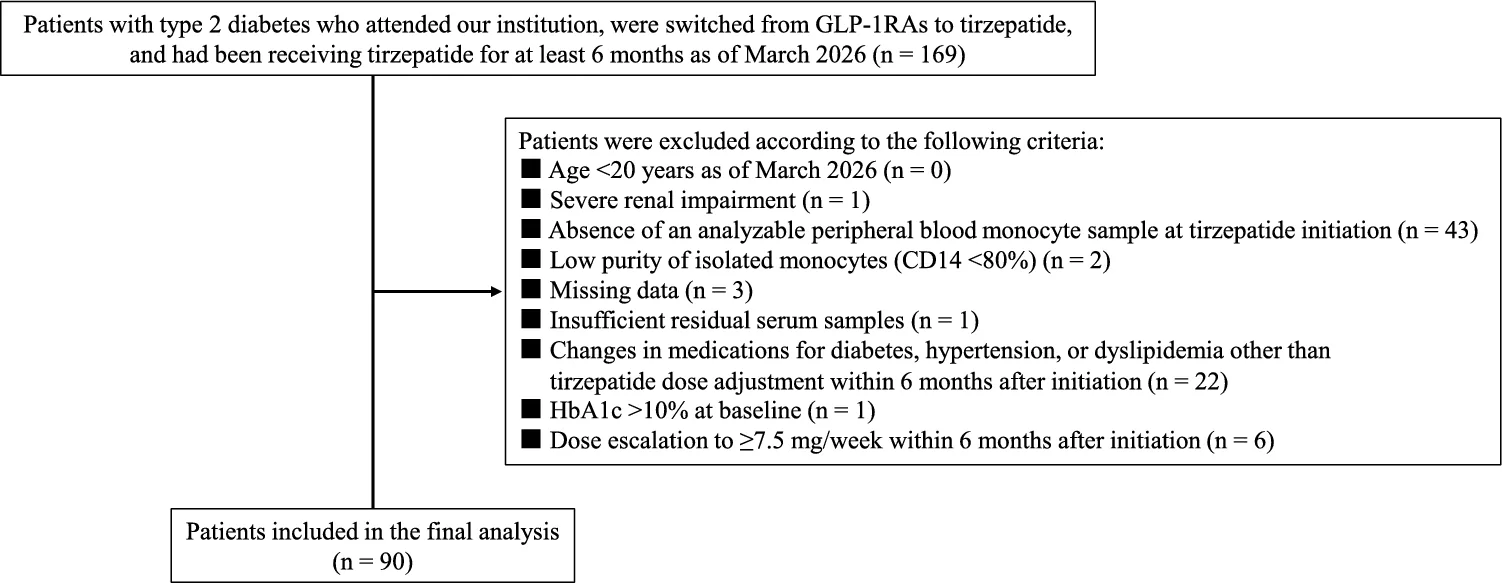
Flowchart showing the participant selection process. The process of selecting study participants is shown. A total of 169 patients with type 2 diabetes who were switched from GLP-1RAs to tirzepatide and received tirzepatide for at least 6 months as of March 2026 were screened. After applying the exclusion criteria, 90 patients were included in the final analysis. GLP-1 RAs, glucagon-like peptide−1 receptor agonists; HbA1c, hemoglobin A1c

This single-center retrospective observational study using prospectively collected peripheral blood monocyte samples included patients with type 2 diabetes who attended the outpatient clinic of Nerima Hikarigaoka Hospital between October 2023 and March 2026. All patients who regularly visited the internal medicine outpatient clinic during the study period and had been switched from GLP-1RAs to tirzepatide were screened for eligibility. Eligible patients had received a GLP-1RA, either as monotherapy or in combination with oral hypoglycemic agents or insulin, for at least 6 months before switching. The GLP-1RAs used before switching were dulaglutide (0.75 mg/week) or semaglutide (1.0 mg/week). Tirzepatide was initiated at 2.5 mg/week and increased to 5 mg/week after 1 month. All included patients had received tirzepatide for at least 6 months as of March 2026. The exclusion criteria were as follows: (1) age < 20 years as of March 2026; (2) severe renal impairment, defined as receipt of renal replacement therapy or estimated glomerular filtration rate < 15 mL/min/1.73 m^2^; (3) absence of an analyzable peripheral blood monocyte sample at tirzepatide initiation; (4) low purity of isolated monocytes (CD14 < 80%); (5) missing data; (6) insufficient residual serum samples; (7) changes in medications for diabetes, hypertension, or dyslipidemia other than tirzepatide dose adjustment within 6 months after initiation; (8) baseline HbA1c >10%; and (9) dose escalation to ≥ 7.5 mg/week within 6 months. Peripheral blood samples for monocyte isolation were prospectively collected at the initiation or modification of incretin-based therapy from consenting patients under an Institutional Review Board-approved prospective observational protocol. Samples for PCR analysis were processed on the same day and stored at − 80 °C. Patients with baseline HbA1c >10% were excluded to reduce potential confounding related to marked hyperglycemia and treatment intensification during follow-up. Of 169 patients switched from GLP-1RAs to tirzepatide, 90 were included in the primary analysis after application of the inclusion and exclusion criteria (Fig. 1). For sensitivity analyses addressing potential selection bias related to treatment course, an expanded cohort additionally included patients who had been excluded from the primary analysis because of tirzepatide dose escalation to ≥ 7.5 mg/week (n = 6), changes in concomitant medications (n = 22), baseline HbA1c > 10% (n = 1), or severe renal impairment (n = 1), provided that an adequate stored monocyte sample was available for *GIPR* mRNA analysis. Thus, the expanded sensitivity cohort comprised 120 participants. All six participants who underwent dose escalation were receiving tirzepatide 10 mg/week at 6 months, and all 22 participants with medication changes had newly initiated an SGLT2 inhibitor during follow-up. Two participants who were unable to tolerate escalation to tirzepatide 5 mg/week had stored total RNA of insufficient quality for reliable PCR analysis and were therefore classified among the patients without analyzable baseline monocyte samples and could not be included in the sensitivity analyses.

### Clinical data collection

**Fig. 2:**
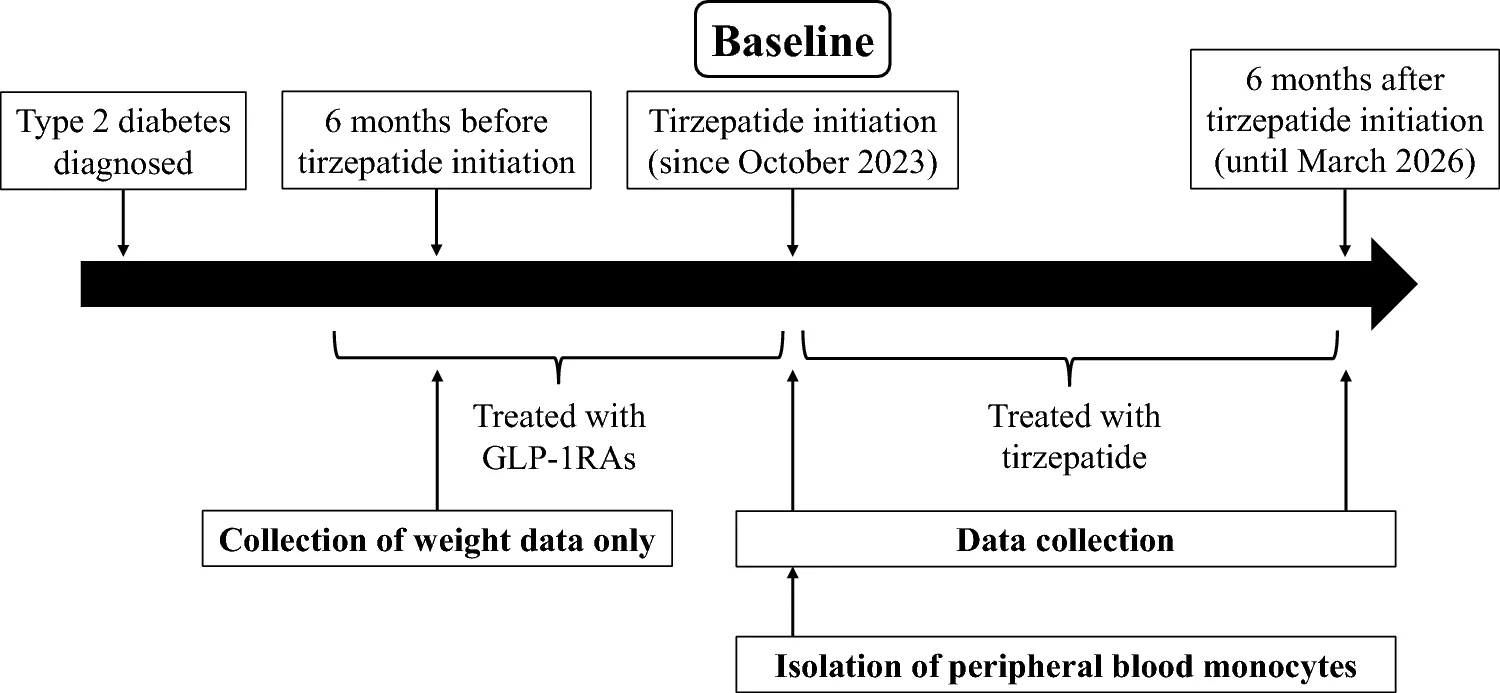
Timeline of this study. Clinical data were collected at baseline, defined as the date of switching from GLP-1RAs to tirzepatide, and at 6 months after tirzepatide initiation. Body weight data from 6 months before tirzepatide initiation were additionally obtained from the medical records. Peripheral blood monocytes were isolated at baseline only. GLP-1 RAs, glucagon-like peptide−1 receptor agonists

The study timeline is illustrated in Fig. 2. Peripheral blood monocytes were collected at baseline, defined as the time of tirzepatide initiation, whereas clinical and laboratory data were obtained at baseline and 6 months after tirzepatide initiation. Data on demographic characteristics, physiological measurements, diabetic complications, laboratory test results, and prescribed medications were collected. For the analysis of body-weight change during the preceding GLP-1RA treatment period, body weight recorded 6 months before tirzepatide initiation was additionally extracted from the electronic medical records. Furthermore, at baseline only, additional peripheral blood was collected, with patient consent, for the isolation of monocytes. All biochemical data were obtained from all participants. Blood samples were collected in the morning after at least 12 h of fasting. HbA1c levels were measured using a latex agglutination method. The FIB-4 index was calculated using the following formula: FIB-4 = (age [years] × aspartate aminotransferase (AST) [U/L]) / (platelet count [× 10^9^/L] × √alanine aminotransferase (ALT) [U/L]), and was extracted from electronic medical records as a marker of liver fibrosis in MASLD [9]. The FIB-4 index was used as a routinely available non-invasive fibrosis-related index for liver fibrosis risk stratification in MASLD [9]. Serum high-sensitivity C-reactive protein (hsCRP) levels were measured using an immunoturbidimetric assay (CRP-Latex; Kamiya Biomedical Company, WA, USA) according to the manufacturer’s protocol.

### Isolation of peripheral blood monocytes and exploratory ex vivo functional assays

Fasting peripheral blood samples were collected in the early morning into 10-mL K2-EDTA Vacutainer tubes. Peripheral blood monocytes were isolated directly from whole blood using the EasySep Direct Human Monocyte Isolation Kit and EasyEights EasySep Magnet (STEMCELL Technologies, Vancouver, Canada), according to the manufacturer’s protocol. This column-free immunomagnetic negative selection method was selected to minimize cellular activation during isolation. Monocyte purity was assessed by flow cytometry using a FACSCelesta (Becton, Dickinson and Company, NJ, USA) with a phycoerythrin-conjugated anti-human CD14 antibody (BioLegend, CA, USA), as previously described [10]. Samples with monocyte purity < 80% were excluded from the analysis; accordingly, data from two subjects (76% and 78% purity, respectively) were excluded. Monocytes were analyzed as a total monocyte population; classical, intermediate, and non-classical monocyte subsets were not separately quantified. Isolated monocytes were resuspended in Roswell Park Memorial Institute (RPMI) 1640 medium (Thermo Fisher Scientific Inc., Waltham, MA, USA) supplemented with 10% fetal bovine serum and 1× antibiotic–antimycotic solution. Cells were seeded at a density of 1 × 10^6^ cells/well in 24-well culture plates (Corning, Tewksbury, MA, USA) and incubated for 24 h at 37 °C in a humidified atmosphere containing 5% CO_2_.

After 24 h of culture, cultured monocytes were pretreated with vehicle or 1 nM human GIP (1–42) (Bachem, Bubendorf, Switzerland) for 30 min, followed by stimulation with vehicle or 10 ng/mL lipopolysaccharide (LPS) for 4 h. For the analysis of culture supernatants, monocytes were stimulated with either vehicle or 10 ng/mL LPS for 24 h instead of 4 h. The cultured monocytes were then harvested, and total RNA was immediately extracted. mRNA expression of interleukin-6 (*IL-6*) and tumor necrosis factor-alpha (*TNFA*) in monocytes and concentrations of IL-6 and TNF-α in the culture supernatants were assessed in unstimulated samples, samples stimulated with LPS alone, and those co-stimulated with LPS and GIP. The concentrations of IL-6 and TNF-α in the culture supernatants were measured using the Quantikine Human IL-6 ELISA Kit and Quantikine Human TNF-α ELISA Kit (R&D Systems, MN, USA), respectively. As an exploratory subset analysis for supporting the validity of the ex vivo GIP stimulation condition, intracellular cyclic adenosine monophosphate (cAMP) levels were measured in monocytes from 23 participants for whom sufficient residual cell samples were available. For this assay, separate wells were prepared under the same ex vivo culture conditions described above. After 24 h of culture, monocytes were incubated with 500 nmol/L 3-isobutyl-1-methylxanthine and then treated with vehicle or 1 nM human GIP (1–42) for 30 min. Cells were immediately harvested after stimulation, and intracellular cAMP concentrations were determined with the cAMP-Screen Direct System (Applied Biosystems, Bedford, MA, USA). Native human GIP (1–42) was used because these exploratory experiments were designed to assess monocyte responses related to the GIP/GIPR axis without concomitant GLP-1R agonism; direct stimulation with GLP-1 or tirzepatide was not performed because of limited monocyte availability. A concentration of 1 nM was selected with reference to a previous study examining GIP responses in human monocytes [11]. Because of the limited number of monocytes available from each participant, a single GIP concentration was used.

### qRT-PCR analysis

Total RNA was extracted from cultured human monocytes using TRIzol reagent (Thermo Fisher Scientific), as previously described [12]. Reverse transcription was performed using random primers (Thermo Fisher Scientific) and ReverTra Ace reverse transcriptase (Toyobo, Osaka, Japan). qRT-PCR was performed using a ViiA 7 Real-Time PCR System and Fast SYBR Green Master Mix (Thermo Fisher Scientific), according to the manufacturer’s protocol. The mRNA expression levels of each gene were normalized to those of actin beta, and relative quantification was performed using the comparative threshold cycle method. qRT-PCR measurements were generally performed in triplicate, and results were accepted when the difference in Ct values among technical replicates was < 0.5 cycles. Primer amplification efficiencies, assessed using standard curves, were within 90–110%. No-template controls and no-reverse-transcriptase controls were included to assess nonspecific amplification and potential genomic DNA contamination. A Ct value of 35 was used as the detection threshold; samples with Ct values > 35 or no detectable amplification were excluded from the expression analysis. All analyzable monocyte samples in the primary cohort showed Ct values below this threshold. Primer sequences are provided in Supplementary Table S1. Ct values for *GIPR*, *GLP-1R*, and *ACTB* are summarized in Supplementary Table S2. Monocyte-derived mRNA expression data were available for all participants.

### Outcome measures

The primary outcomes were percentage change in body weight and change in the FIB-4 index at 6 months after initiation of tirzepatide. Percentage change in body weight was calculated as follows: [(body weight at 6 months − baseline body weight) / baseline body weight] × 100, with negative values indicating weight reduction. Change in the FIB-4 index was calculated as the difference between the values at 6 months and baseline (FIB-4 index at 6 months − baseline FIB-4 index), with negative values indicating a reduction in the FIB-4 index. For the analysis of body-weight change during the preceding GLP-1RA treatment period, percentage change in body weight during the preceding 6 months of GLP-1RA treatment was calculated as [(body weight at tirzepatide initiation − body weight 6 months before tirzepatide initiation) / body weight 6 months before tirzepatide initiation] × 100.

### Statistical analyses

Statistical analyses were performed using Prism 9 (GraphPad Software Inc., San Diego, CA, USA). Continuous variables with a normal distribution were analyzed using Student’s *t*-test, whereas non-normally distributed variables were analyzed using the Mann–Whitney *U* test or Wilcoxon signed-rank test, as appropriate. Categorical variables were analyzed using the chi-square test or Fisher’s exact test. Associations between variables were assessed using Spearman’s rank correlation coefficient. To examine whether monocyte *GIPR* mRNA expression at tirzepatide initiation reflected a general tendency toward weight change during incretin therapy, its association with percentage change in body weight during the preceding 6 months of GLP-1RA treatment was evaluated in all 90 participants using Spearman’s rank correlation analysis. For the exploratory correlations between monocyte *GIPR* or *GLP-1R* mRNA expression and the changes in clinical parameters after 6 months of tirzepatide treatment, the Benjamini–Hochberg procedure was applied across all comparisons to control the false discovery rate (FDR). FDR-adjusted P values are reported as q values, with q <0.05 considered statistically significant. For multiple linear regression analyses, covariates were selected a priori based on clinical relevance and potential confounding to reduce the risk of model overfitting. For the analysis with percentage change in body weight at 6 months as the dependent variable, the multivariable model included baseline monocyte *GIPR* expression, age, duration of diabetes, body mass index (BMI), HbA1c, and the type of GLP-1RA used before switching to tirzepatide. For the analysis with change in the FIB-4 index at 6 months as the dependent variable, the multivariable model 1 included baseline monocyte *GIPR* expression, duration of diabetes, BMI, HbA1c, baseline FIB-4 index, and the type of GLP-1RA used before switching to tirzepatide. Baseline FIB-4 index was included instead of its individual components to avoid excessive adjustment and collinearity. The multivariable model 2 further included percentage change in body weight during the 6-month treatment period. In the expanded sensitivity cohort (n=120), the corresponding models additionally included tirzepatide dose at 6 months and initiation of an SGLT2 inhibitor during follow-up. To assess potential selection bias related to monocyte sample availability, baseline clinical characteristics were compared between the 90 participants included in the primary analysis and the 43 patients without analyzable baseline monocyte samples. Non-normally distributed continuous variables were log-transformed before inclusion in the regression models. For naturally log-transformed *GIPR* expression, the estimated difference in the dependent variable associated with a doubling of *GIPR* expression was calculated as β × ln(2). Regression assumptions were assessed using Shapiro–Wilk tests and Q–Q plots for residual normality, residual-versus-fitted plots and the Breusch–Pagan test for homoscedasticity, Cook’s distance for influential observations, and variance inflation factors for multicollinearity. No substantial violations of the assumptions of the multiple linear regression model were identified. A two-sided P value <0.05 was considered statistically significant.

## Results

### Baseline characteristics and monocyte *GIPR* mRNA expression

The baseline characteristics of the 90 participants included in the primary analysis are presented in Table 1. GIPR and GLP-1R transcripts were detectable in all analyzable monocyte samples, with Ct values below the predefined detection threshold of 35 (Supplementary Table S2). Spearman’s rank correlation analysis was performed to assess associations between monocyte incretin receptor expression and baseline clinical parameters (Supplementary Table S3). Monocyte *GIPR* mRNA expression was significantly inversely correlated with age and duration of diabetes, and positively correlated with AST levels and the FIB-4 index. In contrast, monocyte *GIPR* mRNA expression was not significantly correlated with BMI or hsCRP at baseline. Monocyte *GLP-1R* mRNA expression was significantly inversely correlated with age and positively correlated with γ-glutamyl transpeptidase (γ-GTP) levels. Monocyte *GIPR* mRNA expression measured at tirzepatide initiation was not significantly associated with percentage change in body weight during the preceding 6 months of GLP-1RA treatment (Supplementary Fig. S1).

**Table 1:** Baseline characteristics of the study participants

	Total cohort (n = 90)
*Clinical characteristics*	
Age (years)	57.5±10.8
Female sex, *n* (%)	44 (49)
Duration of diabetes (years)	11 (3–21)
Current smoker (*n*, %)	26 (29)
Body weight (kg)	74.9±15.4
BMI (kg/m^2^)	27.6±4.6
Waist circumference (cm)	89.7±7.3
*Complications of diabetes (n, %)*	
Neuropathy	45 (50)
Retinopathy	33 (37)
Nephropathy	55 (61)
Atherosclerotic diseases	22 (24)
*Physiological testing*	
SBP (mmHg)	135±10
DBP (mmHg)	71±6
*Laboratory parameters*	
HbA1c (%)	8.05 (7.50–9.10)
TG (mg/dL)	246 (186–327)
HDL-C (mg/dL)	44 (40–49)
LDL-C (mg/dL)	110 (95–120)
AST (IU/L)	30 (19–36)
ALT (IU/L)	39 (29–47)
γ-GTP (IU/L)	54 (34–71)
UA (mg/dL)	5.9 (4.3–7.1)
UACR (mg/gCr)	39.9 (17.2–76.6)
eGFR (mL/min/1.73m^2^)	70.8 (60.4–80.0)
hsCRP (mg/L)	2.9 (2.5–3.6)
FIB-4 index	1.18 (0.94–1.56)
*GIPR* expression in monocytes	0.008 (0.001–0.016)
*GLP-1R* expression in monocytes	0.004 (0.002–0.007)
*Medications (n, %)*	
Insulin	18 (20)
Sulfonylureas	9 (10)
Glinides	8 (9)
GLP-1RAs (semaglutide)	45 (50)
Metformin	77 (86)
TZDs	7 (8)
Alpha-GIs	5 (6)
SGLT2 inhibitors	54 (60)
ARBs	42 (47)
CCBs	26 (29)
Statins	57 (63)
Fibrates	38 (42)
UA lowering agents	22 (24)

Data are expressed as the mean ± standard deviation, median (interquartile range), or number (%). *ALT* alanine aminotransferase, *ARBs* angiotensin receptor blockers, *AST* aspartate aminotransferase, *BMI* body mass index, *CCBs* calcium channel blockers, *DBP* diastolic blood pressure, *eGFR* estimated glomerular filtration rate, *FIB-4* fibrosis 4, *γ-GTP* γ-glutamyl transpeptidase, *GIPR* glucose-dependent insulinotropic polypeptide receptor, *GIs* glycosidase inhibitors, *GLP-1R* glucagon-like peptide-1 receptor, *GLP-1RAs* glucagon-like peptide-1 receptor agonists, *HbA1c* hemoglobin A1c, *HDL-C* high-density lipoprotein cholesterol, *hsCRP* high-sensitivity C-reactive protein, *LDL-C* low-density lipoprotein cholesterol, *SBP* systolic blood pressure, *SGLT2* sodium-glucose cotransporter 2, *TG* triglycerides, *TZDs* thiazolidinediones, *UA* uric acid, *UACR* urine albumin-to-creatinine ratio

### hanges in clinical parameters and exploratory correlation analyses

After 6 months of tirzepatide treatment, body weight, BMI, waist circumference, systolic blood pressure, HbA1c, triglycerides (TG), AST, ALT, uric acid, UACR, hsCRP, and the FIB-4 index were significantly reduced, whereas high-density lipoprotein cholesterol was significantly increased (Table 2). The FIB-4 index decreased from 1.18 to 0.94 together with significant reductions in AST and ALT. To examine whether monocyte incretin receptor expression was associated with changes in clinical and metabolic parameters during tirzepatide treatment, Spearman’s rank correlation analyses were performed (Table 3). Monocyte *GIPR* mRNA expression was significantly associated with changes in percentage body weight, BMI, waist circumference, HbA1c, TG, AST, ALT, UACR, eGFR, hsCRP, and the FIB-4 index. Monocyte *GLP-1R* mRNA expression was significantly associated with change in HbA1c. All correlations that were significant using the unadjusted P values remained significant after Benjamini–Hochberg FDR adjustment (q <0.05).

**Table 2:** Changes in clinical parameters from baseline to 6 months after initiation of tirzepatide

	Overall (*n* = 90)		
	Baseline	After	*P-*value
Body weight (kg)	74.9 ± 15.4	72.3 ± 14.0	< 0.001
BMI (kg/m^2^)	27.6 ± 4.6	26.7 ± 4.2	< 0.001
Waist circumference (cm)	89.7 ± 7.3	85.0 ± 6.3	< 0.001
SBP (mmHg)	135 ± 10	130 ± 9	< 0.001
DBP (mmHg)	71 ± 6	70 ± 6	0.165
HbA1c (%)	8.05 (7.50–9.10)	7.15 (5.90–8.20)	< 0.001
TG (mg/dL)	246 (186–327)	198 (153–269)	0.033
HDL-C (mg/dL)	44 (40–49)	47 (42–52)	< 0.001
LDL-C (mg/dL)	110 (95–120)	108 (98–123)	0.419
AST (IU/L)	30 (19–36)	25 (18–31)	< 0.001
ALT (IU/L)	39 (29–47)	28 (19–39)	< 0.001
γ-GTP (IU/L)	54 (34–71)	52 (30–73)	0.107
UA (mg/dL)	5.9 (4.3–7.1)	5.1 (3.3–6.8)	0.025
UACR (mg/gCr)	39.9 (17.2–76.6)	29.3 (13.4–52.3)	0.004
eGFR (mL/min/1.73m^2^)	70.8 (60.4–80.0)	66.3 (60.4–78.2)	0.352
hsCRP (mg/L)	2.9 (2.5–3.6)	2.0 (1.4–2.8)	< 0.001
FIB-4 index	1.18 (0.94–1.56)	0.94 (0.75–1.16)	< 0.001

Data are expressed as the mean ± standard deviation, or the median (interquartile range), and are compared using the paired *t*-test or the Wilcoxon signed-rank test. *ALT* alanine aminotransferase, *AST* aspartate aminotransferase, *BMI* body mass index, *DBP* diastolic blood pressure, *eGFR* estimated glomerular filtration rate, *FIB-4* fibrosis 4, *γ-GTP* γ-glutamyl transpeptidase, *HbA1c* hemoglobin A1c, *HDL-C* high-density lipoprotein cholesterol, *hsCRP* high-sensitivity C-reactive protein, *LDL-C* low-density lipoprotein cholesterol, *SBP* systolic blood pressure, *TG* triglycerides, *UA* uric acid, *UACR* urine albumin-to-creatinine ratio

**Table 3:** Correlation analysis of monocyte GIP receptor and GLP-1 receptor mRNA expression levels with changes in clinical parameters

	*GIPR* expression in monocytes			*GLP-1R* expression in monocytes		
	*ρ*	*P*-value	q-value (FDR)	*ρ*	*P*-value	q-value (FDR)
Percentage change in body weight (%)	− 0.614	< 0.001	< 0.001	− 0.179	0.091	0.205
ΔBMI (kg/m^2^)	− 0.600	< 0.001	< 0.001	− 0.187	0.078	0.192
ΔWaist circumference (cm)	− 0.398	< 0.001	0.001	− 0.009	0.933	0.962
ΔSBP (mmHg)	− 0.096	0.367	0.567	− 0.040	0.707	0.777
ΔDBP (mmHg)	− 0.170	0.109	0.219	− 0.043	0.690	0.777
ΔHbA1c (%)	− 0.389	< 0.001	0.001	− 0.287	0.006	0.016
ΔTG (mg/dL)	− 0.351	< 0.001	0.003	0.108	0.311	0.508
ΔHDL-C (mg/dL)	0.176	0.098	0.219	0.136	0.201	0.344
ΔLDL-C (mg/dL)	− 0.069	0.519	0.678	− 0.005	0.963	0.962
ΔAST (IU/L)	− 0.324	0.002	0.006	− 0.167	0.116	0.221
ΔALT (IU/L)	− 0.457	< 0.001	< 0.001	− 0.070	0.512	0.678
Δγ-GTP (IU/L)	− 0.198	0.061	0.159	− 0.043	0.685	0.777
ΔUA (mg/dL)	0.087	0.414	0.604	− 0.035	0.741	0.777
ΔUACR (mg/gCr)	− 0.366	< 0.001	0.002	− 0.027	0.798	0.865
ΔeGFR (mL/min/1.73 m^2^)	0.304	0.004	0.012	0.073	0.493	0.678
ΔhsCRP (mg/L)	− 0.532	< 0.001	< 0.001	− 0.057	0.595	0.749
ΔFIB-4 index	− 0.505	< 0.001	< 0.001	− 0.140	0.188	0.336

Spearman’s rank correlation analysis was performed. The change in each parameter (Δ) was calculated by subtracting the baseline value from the value obtained at 6 months after the initiation of tirzepatide. Percentage change in body weight (%) was calculated as follows: [(body weight at 6 months − baseline body weight) / baseline body weight] × 100. Negative values indicate weight reduction. To account for multiple comparisons, the Benjamini–Hochberg procedure was applied across all 34 correlation tests presented in this table to control the false discovery rate (FDR). FDR-adjusted *P* values are presented as q values, with q < 0.05 considered statistically significant. *ALT* alanine aminotransferase, *AST* aspartate aminotransferase, *BMI* body mass index, *DBP* diastolic blood pressure, *eGFR* estimated glomerular filtration rate, *FIB-4* fibrosis 4, *GIPR* glucose-dependent insulinotropic polypeptide receptor, *GLP-1R* glucagon-like peptide-1 receptor, *γ-GTP* γ-glutamyl transpeptidase, *HbA1c* hemoglobin A1c, *HDL-C* high-density lipoprotein cholesterol, *hsCRP* high-sensitivity C-reactive protein, *LDL-C* low-density lipoprotein cholesterol, *SBP* systolic blood pressure, *ρ* Spearman''s correlation coefficient, *TG* triglycerides, *UA* uric acid, *UACR* urine albumin-to-creatinine ratio

### Exploratory ex vivo findings

**Fig. 3:**
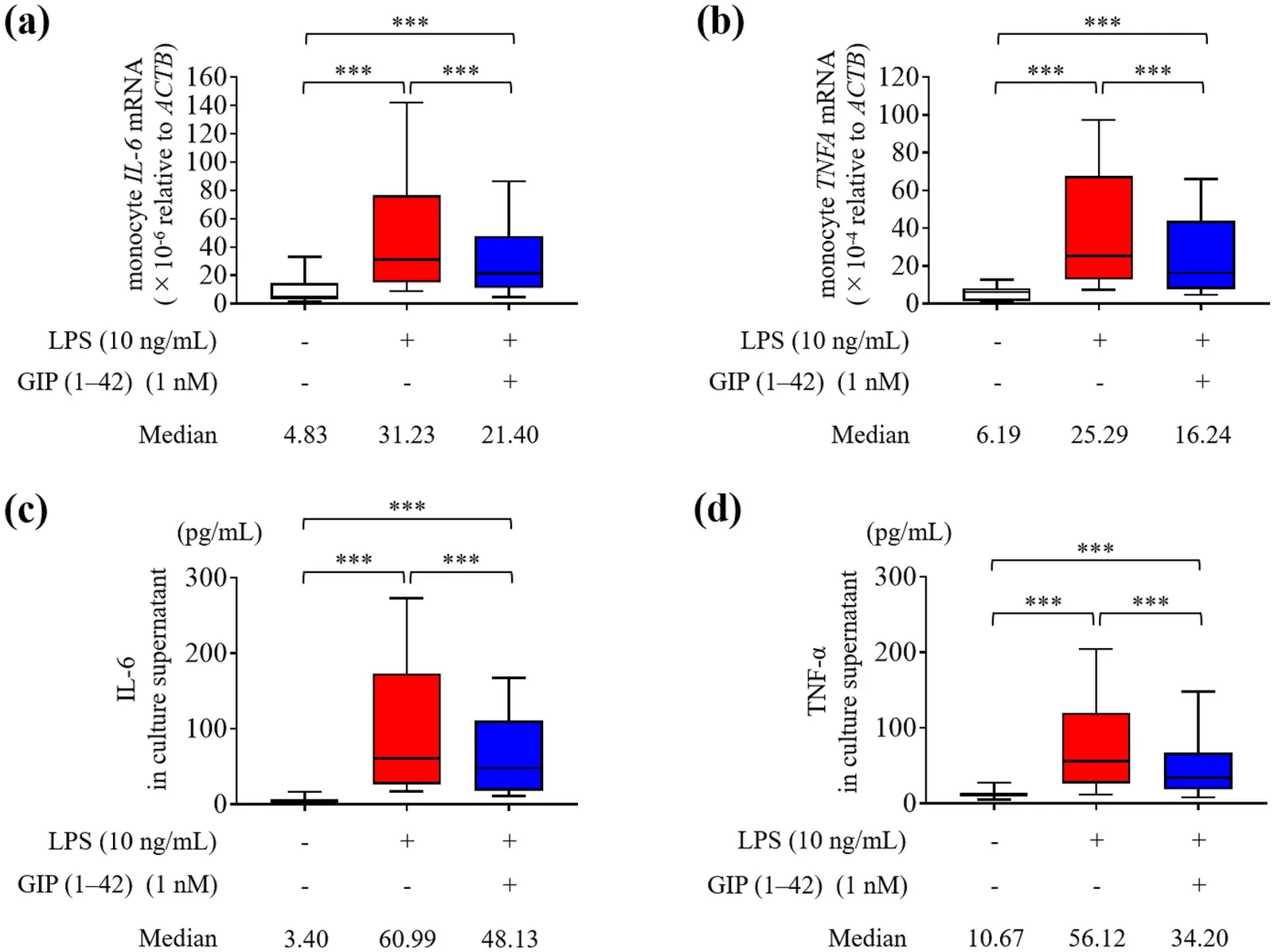
Ex vivo effects of GIP on LPS-induced IL-6 and TNF-α responses in peripheral blood monocytes. Cultured monocytes were treated with LPS at a concentration of 10 ng/mL for 4 h, with or without a pretreatment of GIP (1–42) at a concentration of 1 nM for 30 min. For the analysis of culture supernatants, monocytes were stimulated with vehicle or 10 ng/mL LPS for 24 h, rather than 4 h, with the same GIP (1–42) pretreatment procedure. Quantitative analyses are shown for the mRNA expression levels of **a** *IL-6* and **b** *TNFA* in monocytes and the concentrations of **c** IL-6 and **d** TNF-α in the culture supernatants (n = 90). Data were analyzed using the Wilcoxon signed-rank test. All data are presented as box-and-whisker plots. The box represents the interquartile range, the horizontal line within the box indicates the median, and the whiskers represent the 10th and 90th percentiles. The median values are indicated at the bottom of each panel. ACTB, actin beta; GIP, glucose-dependent insulinotropic polypeptide; IL-6, interleukin-6; LPS, lipopolysaccharide; TNFA, tumor necrosis factor-alpha. ***P < 0.001

**Fig. 4:**
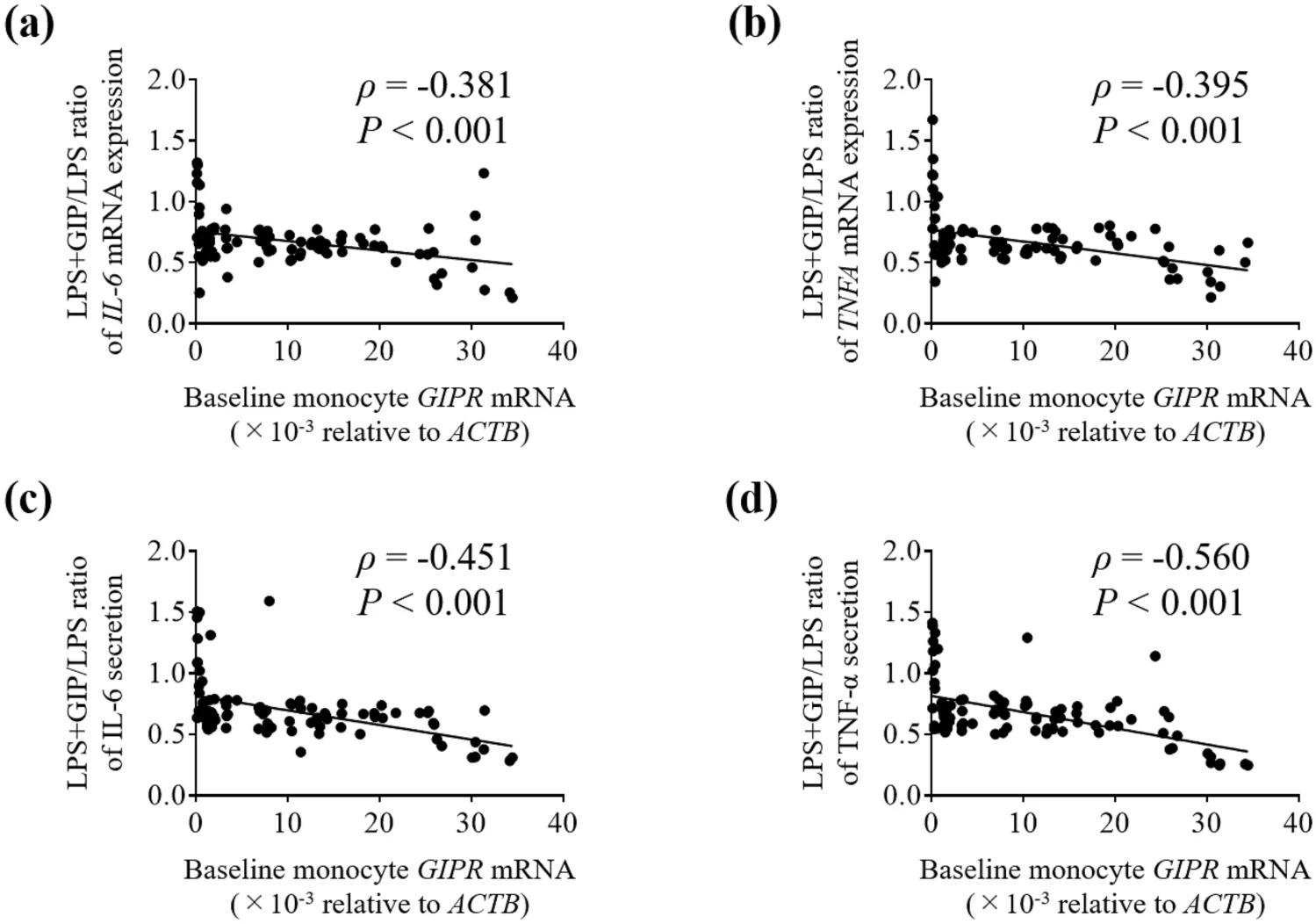
Associations between baseline monocyte *GIPR* mRNA expression and GIP-mediated suppression of LPS-induced inflammatory responses. The correlations between baseline monocyte *GIPR* mRNA expression levels in unstimulated samples and the ratios of **a** *IL-6* and **b** *TNFA* mRNA expression in monocytes and the ratios of **c** IL-6 and **d** TNF-α concentrations in the culture supernatants after GIP treatment were evaluated using Spearman’s rank correlation analysis. The ratio of each parameter after GIP treatment was calculated as the value in the LPS plus GIP group divided by the value in the LPS-alone group. Lower values indicate greater suppression of LPS-induced inflammatory responses by GIP. ACTB, actin beta; GIP, glucose-dependent insulinotropic polypeptide; GIPR, glucose-dependent insulinotropic polypeptide receptor; IL-6, interleukin-6; LPS, lipopolysaccharide; ρ, Spearman''s correlation coefficient; TNFA, tumor necrosis factor-alpha

Given the observed associations between baseline monocyte *GIPR* mRNA expression and changes in metabolic and inflammation-related parameters, in all 90 participants, we performed an exploratory ex vivo stimulation assay. LPS-induced mRNA expression of *IL-6* and *TNFA* was significantly suppressed in the LPS plus GIP group compared with the LPS-alone group (Fig. 3). Consistently, the concentrations of IL-6 and TNF-α in the culture supernatant were also significantly lower in the LPS plus GIP group than in the LPS-alone group (Fig. 3). We further examined whether baseline monocyte *GIPR* mRNA expression was associated with the degree of GIP-mediated suppression of LPS-induced inflammatory responses. For this analysis, the relative response after GIP treatment was calculated as the ratio of the value in the LPS plus GIP group to that in the LPS-alone group. The ratios of *IL-6* and *TNFA* mRNA expression, as well as the ratios of IL-6 and TNF-α concentrations in the culture supernatants, were significantly inversely correlated with baseline monocyte *GIPR* mRNA expression levels in unstimulated samples (Fig. 4). In addition, we performed an exploratory subset analysis of the cAMP response to short-term GIP stimulation in peripheral blood monocytes from 23 participants with sufficient residual monocyte samples. Intracellular cAMP levels were significantly increased 30 min after native GIP stimulation compared with vehicle stimulation (Supplementary Fig. S2). Furthermore, baseline monocyte *GIPR* mRNA expression was significantly positively correlated with the magnitude of GIP-induced cAMP elevation in this subset (Supplementary Fig. S2). Taken together, native GIP suppressed LPS-induced inflammatory responses in peripheral blood monocytes.

### Multivariable analyses of body-weight change

In simple linear regression analysis, baseline monocyte *GIPR* mRNA expression was significantly associated with percentage change in body weight (Supplementary Table S4). This association remained significant after adjustment for age and sex and in the prespecified multivariable model including age, duration of diabetes, baseline BMI, baseline HbA1c, and previous GLP-1RA type (Table 4). In the fully adjusted model, the unstandardized coefficient for naturally log-transformed monocyte *GIPR* expression was − 0.518 (*P* = 0.002). A doubling of baseline monocyte *GIPR* mRNA expression therefore corresponded to an estimated 0.36-percentage-point lower percentage change in body weight, equivalent to approximately 0.36 percentage points greater weight reduction after adjustment for the other covariates.

**Table 4:** Multiple linear regression model examining the association between baseline monocyte GIP receptor mRNA expression and percentage change in body weight after 6 months of tirzepatide treatment

Dependent variable: Percentage change in body weight			
	β coefficient	95% CI	*P*-value
*Age- and sex-adjusted model*	(Adjusted *R*^2^ = 0.426)		
*GIPR* expression in monocytes ^†^	− 0.583	− 0.906 to − 0.259	< 0.001
Age	0.113	0.065 to 0.162	< 0.001
Female sex	0.435	− 0.469 to 1.339	0.342
*Multiple regression model*	(Adjusted *R*^2^ = 0.488)		
*GIPR* expression in monocytes ^†^	− 0.518	− 0.847 to − 0.189	0.002
Age	0.071	0.019 to 0.122	0.008
Duration of diabetes ^†^	0.426	− 0.088 to 0.940	0.103
BMI	− 0.091	− 0.193 to 0.012	0.083
HbA1c ^†^	3.331	− 0.498 to 7.160	0.087
Previous GLP-1RA type: semaglutide	0.746	− 0.173 to 1.666	0.110

All independent variables corresponded to the values obtained at baseline. The model included covariates selected a priori based on clinical relevance and potential confounding. Percentage weight change (%) was calculated as follows: [(body weight at 6 months − baseline body weight) / baseline body weight] × 100. Negative values indicate weight reduction. *BMI* body mass index, *CI* confidence interval, *GIPR* glucose-dependent insulinotropic polypeptide receptor, *GLP-1RAs* glucagon-like peptide-1 receptor agonists, *HbA1c* hemoglobin A1c, *β* partial regression coefficient

^†^Log-transformed using the natural logarithm

### Multivariable analyses of FIB-4 index change

Baseline monocyte *GIPR* mRNA expression was significantly associated with change in the FIB-4 index in simple linear regression analysis (Supplementary Table S5). In Model 1, adjusted for duration of diabetes, baseline BMI, HbA1c, baseline FIB-4 index, and previous GLP-1RA type, monocyte *GIPR* mRNA expression remained significantly associated with change in the FIB-4 index (Supplementary Table S6). When percentage change in body weight was additionally included in Model 2, baseline monocyte *GIPR* mRNA expression remained significantly associated with change in the FIB-4 index (Supplementary Table S6).

### Sensitivity analyses and assessment of selection bias

In the expanded sensitivity cohort of 120 participants, baseline monocyte *GIPR* mRNA expression remained significantly associated with percentage body-weight change after additional adjustment for tirzepatide dose at 6 months and SGLT2 inhibitor initiation during follow-up, along with the covariates included in the primary analysis (Supplementary Table S7). Baseline monocyte *GIPR* mRNA expression also remained significantly associated with change in the FIB-4 index in the corresponding sensitivity model and after further adjustment for percentage change in body weight (Supplementary Table S8). To assess potential selection bias related to baseline monocyte sample availability, baseline clinical characteristics were compared between the 90 participants included in the primary analysis and the 43 patients without analyzable baseline monocyte samples. No statistically significant differences were observed in the assessed baseline characteristics between the two groups (Supplementary Table S9).

## Discussion

In this study, baseline *GIPR* mRNA expression in peripheral blood monocytes was associated with both weight reduction and reduction in the FIB-4 index after switching from GLP-1RAs to tirzepatide. These associations remained significant in multivariable analyses, and the association between baseline monocyte *GIPR* mRNA expression and change in the FIB-4 index persisted after additional adjustment for percentage body-weight change. The associations with body-weight and FIB-4 index changes were also maintained in an expanded sensitivity cohort. In addition, exploratory ex vivo functional data showed that native GIP suppressed LPS-induced IL-6 and TNF-α responses in monocytes, and that the degree of this suppression was associated with baseline monocyte *GIPR* mRNA expression. Collectively, these findings suggest that baseline monocyte *GIPR* mRNA expression may represent an exploratory cell-specific transcriptomic feature associated with interindividual variation in clinical changes after switching to tirzepatide. However, because the study was observational, these findings should not be interpreted as evidence that monocyte GIPR expression determines responsiveness to tirzepatide.

The observed association between monocyte *GIPR* mRNA expression and favorable changes in metabolic parameters is consistent with previous evidence linking the GIP/GIPR axis to obesity and insulin resistance. Disruption of GIPR signaling in adipose tissue has been associated with impaired metabolic homeostasis, whereas preserved GIPR expression has been linked to more favorable metabolic regulation [13]. Although monocyte *GIPR* mRNA expression cannot be assumed to represent systemic GIPR activity or receptor function in adipose tissue, liver, or other metabolic organs, the present findings raise the possibility that circulating monocytes capture biologically relevant interindividual variation in the GIP/GIPR-related metabolic milieu that is not apparent from conventional clinical measurements alone. Baseline monocyte *GIPR* mRNA expression was not significantly correlated with BMI or hsCRP. Because adipose tissue macrophage infiltration is a local process [14] and hsCRP is a systemic inflammatory marker, neither necessarily reflects monocyte-specific receptor expression or GIP responsiveness. Thus, monocyte *GIPR* mRNA expression may not simply be a surrogate for adiposity or systemic inflammatory burden. From a translational perspective, peripheral blood monocytes are readily accessible compared with adipose or hepatic tissue and can be sampled repeatedly. If validated prospectively, monocyte *GIPR* mRNA expression could be evaluated as one component of future treatment-stratification strategies. However, predictive performance, clinically useful cutoffs, and independent validation were not assessed in the present study.

Whether the observed association is specific to tirzepatide is an important consideration. Monocyte *GIPR* mRNA expression measured at tirzepatide initiation was not significantly associated with body-weight change during the preceding 6 months of GLP-1RA treatment, whereas it was associated with subsequent body-weight change after switching to tirzepatide. This finding argues against the simple interpretation that higher GIPR expression merely identifies individuals with a general tendency to lose more weight during incretin-based treatment. However, GIPR expression was measured only at the time of switching, and the study did not include a parallel comparator group continuing GLP-1RA therapy. Therefore, the present analysis cannot establish that monocyte *GIPR* mRNA expression specifically reflects responsiveness to tirzepatide or its dual GIPR/GLP-1R agonist mechanism. Human genetic evidence also indicates that GIPR biology is complex. Individuals carrying GIPR loss-of-function variants have been reported to have lower BMI, lower body fat percentage, and a leaner phenotype [15]. Lifelong systemic reduction of GIPR function is biologically distinct from pharmacological dual incretin receptor agonism and from cell-specific variation in *GIPR* transcript abundance in circulating monocytes. Accordingly, the present findings should be interpreted as evidence of biologically relevant heterogeneity associated with clinical changes after switching therapy rather than as evidence that greater systemic GIPR activity directly causes greater weight reduction.

Mechanistically, accumulating evidence suggests that GIPR signaling in myeloid cells regulates inflammation and energy homeostasis. Impaired GIPR signaling in myeloid cells has been associated with enhanced S100A8/A9-related inflammation, obesity, insulin resistance, and hepatic steatosis [8], whereas GIPR signaling in immune cells has been reported to support metabolically favorable immune responses in adipose tissue [16]. The present exploratory ex vivo findings are consistent with this biological framework. Native GIP suppressed LPS-induced IL-6 and TNF-α responses in peripheral blood monocytes, and the magnitude of this suppression was associated with baseline monocyte *GIPR* mRNA expression. GIP stimulation also increased intracellular cAMP in the exploratory subset, and baseline *GIPR* mRNA expression was associated with the magnitude of this response. Because IL-6 and TNF-α are representative mediators of metabolic inflammation [17, 18], these observations provide supportive functional context for the clinical associations. Circulating monocytes also serve as precursors of tissue macrophages [19], and experimental studies have reported that tirzepatide reduces adipose tissue inflammation and macrophage infiltration [20], while monocyte and macrophage phenotypes have been associated with responses to incretin-based therapies [21]. Nevertheless, these observations do not establish a direct receptor-dependent mechanism. GIPR blockade or genetic inhibition was not performed, and the experiments used native GIP rather than tirzepatide. Therefore, the ex vivo findings should be regarded as exploratory observations related to the GIP/GIPR axis rather than direct mechanistic evidence of tirzepatide action.

The association between monocyte *GIPR* mRNA expression and change in the FIB-4 index may also have biological relevance, although it requires cautious interpretation. Recruitment of circulating monocytes and their differentiation into macrophages have been implicated in liver inflammation and fibrosis [22], suggesting a possible link between monocyte-related immunometabolic status and liver-related non-invasive indices. However, FIB-4 index is calculated from age, AST, ALT, and platelet count, and the observed 6-month reduction occurred together with substantial decreases in aminotransferase levels. Therefore, the change may have partly reflected alterations in these biochemical components rather than structural regression of liver fibrosis. In addition, the median baseline FIB-4 index was relatively low, and direct fibrosis assessments using transient elastography, magnetic resonance-based imaging, or liver histology were not available. Although baseline monocyte *GIPR* mRNA expression remained significantly associated with FIB-4 index change after additional adjustment for percentage body-weight change, body-weight change is a post-baseline variable that may lie on the causal pathway. Thus, this analysis does not establish a weight-independent hepatic effect or an antifibrotic mechanism. The finding should therefore be interpreted as an association with change in a non-invasive fibrosis-related index rather than evidence of histological fibrosis regression.

Several limitations should be acknowledged. First, this was a single-center retrospective observational study using prospectively collected peripheral blood monocyte samples. Although the main associations remained significant in expanded sensitivity analyses and measured baseline characteristics did not differ significantly between participants included in the primary analysis and those without analyzable baseline monocyte samples, selection bias and residual confounding cannot be completely excluded. Second, monocyte GIPR expression was assessed only at the mRNA level and at a single baseline time point; protein abundance, cell-surface receptor expression, longitudinal changes, and within-individual reproducibility were not evaluated. Third, monocytes were analyzed as a total population, and classical, intermediate, and non-classical monocyte subsets were not separately quantified; differences in subset composition may therefore have contributed to interindividual variation in *GIPR* mRNA expression. Fourth, circulating monocytes may not fully reflect tissue-resident macrophages or GIPR biology in adipose tissue, liver, or other metabolic organs. Fifth, the exploratory ex vivo experiments were limited by the absence of direct tirzepatide stimulation, dose–response assessment, receptor-specific blockade, and broader functional analyses. Finally, liver-related outcomes were assessed using the FIB-4 index without direct imaging or histological evaluation. Future prospective studies should include comparator treatment groups, longitudinal and subset-specific monocyte sampling, protein-level and receptor-specific functional validation, and direct assessment of liver fibrosis.

In conclusion, higher baseline *GIPR* mRNA expression in peripheral blood monocytes was associated with greater reductions in body weight and the FIB-4 index after switching from GLP-1RAs to tirzepatide. Together with existing evidence linking the GIP/GIPR axis to metabolic regulation and myeloid-cell immunometabolism, these findings suggest that monocyte *GIPR* mRNA expression may capture biologically relevant interindividual heterogeneity and may warrant further investigation as an accessible exploratory marker for treatment stratification. However, the present study does not establish tirzepatide specificity, a causal role of monocyte GIPR signaling, or structural liver fibrosis regression, and prospective validation is required before clinical application can be considered.

## Supplementary Information

Below is the link to the electronic supplementary material.

Supplementary file1 (DOCX 115 kb)

## Data Availability

The datasets analyzed during the current study are not publicly available because they contain potentially identifiable clinical information, but are available from the corresponding author on reasonable request and with appropriate institutional approval.
