# Supplementary material for: Baseline monocyte GIP receptor mRNA expression is associated with reductions in body weight and FIB-4 index after switching to tirzepatide in patients with type 2 diabetes
Source: Diabetol Int. 2026 Oct 31;18(1):4. doi: 10.1007/s13340-026-00940-3 (PMC13723467; doi:10.1007/s13340-026-00940-3)
Supplement: Supplementary file 1 [file 13340_2026_940_MOESM1_ESM.docx]

**SUPPLEMENTARY TABLES**

**Supplementary Table S1. qRT-PCR primer sequences used in the study**

| Gene | Forward primer sequence (5' -3') | Reverse primer sequence (5' -3') |
| --- | --- | --- |
| GIPR | TGGCATTCTCCTGTCCAAGCTG | GTTCCTCTGTCACGGGAGCAAA |
| GLP-1R | CTACGCACTCTCCTTCTCTGCT | CGGACAATGCTCGCAGGATGAA |
| IL-6 | ACTCACCTCTTCAGAACGAATTG | CCATCTTTGGAAGGTTCAGGTTG |
| TNFA | CTCTTCTGCCTGCTGCACTTTG | ATGGGCTACAGGCTTGTCACTC |
| ACTB | GGACTTCGAGCAAGAGATGG | AGCACTGTGTTGGCGTACAG |

ACTB, actin beta; GIPR, glucose-dependent insulinotropic polypeptide receptor; GLP-1R, glucagon-like peptide-1 receptor; IL-6, interleukin-6; qRT-PCR, quantitative real-time polymerase chain reaction; TNFA, tumor necrosis factor-alpha.

**Supplementary Table S2. Ct values for GIP receptor, GLP-1 receptor, and ACTB genes in peripheral blood monocytes**

| Gene | Ct value, median (IQR) | Ct range | Detection rate |
| --- | --- | --- | --- |
| GIPR | 23.390 (22.276–25.951) | 21.046–29.912 | 90/90 |
| GLP-1R | 24.375 (23.323–26.042) | 20.638–29.979 | 90/90 |
| ACTB | 16.387 (16.030–16.710) | 15.306–16.983 | 90/90 |

ACTB, actin beta; IQR, interquartile range; GIPR, glucose-dependent insulinotropic polypeptide receptor; GLP-1R, glucagon-like peptide-1 receptor; qRT-PCR, quantitative real-time polymerase chain reaction.

**Supplementary Table S3. Correlation analysis of monocyte GIP receptor and GLP-1 receptor mRNA expression levels with baseline clinical parameters**

|  | *GIPR* expression in monocytes | | *GLP-1R* expression in monocytes | |
| --- | --- | --- | --- | --- |
|  | *ρ* | *P*-value | *ρ* | *P*-value |
| Age (years) | -0.497 | <0.001 | -0.294 | 0.005 |
| Duration of diabetes (years) | -0.558 | <0.001 | -0.178 | 0.093 |
| Body weight (kg) | 0.170 | 0.110 | -0.014 | 0.898 |
| BMI (kg/m^2^) | 0.147 | 0.167 | 0.066 | 0.536 |
| Waist circumference (cm) | 0.170 | 0.108 | -0.033 | 0.759 |
| SBP (mmHg) | 0.041 | 0.702 | -0.063 | 0.558 |
| DBP (mmHg) | 0.039 | 0.715 | -0.069 | 0.517 |
| HbA1c (%) | 0.163 | 0.126 | -0.073 | 0.496 |
| TG (mg/dL) | 0.077 | 0.472 | -0.070 | 0.514 |
| HDL-C (mg/dL) | -0.034 | 0.750 | -0.083 | 0.436 |
| LDL-C (mg/dL) | 0.167 | 0.116 | 0.012 | 0.910 |
| AST (IU/L) | 0.328 | 0.002 | 0.103 | 0.334 |
| ALT (IU/L) | 0.159 | 0.135 | 0.189 | 0.074 |
| γ-GTP (IU/L) | -0.196 | 0.064 | 0.259 | 0.014 |
| UA (mg/dL) | 0.031 | 0.773 | -0.099 | 0.355 |
| UACR (mg/gCr) | 0.058 | 0.584 | 0.008 | 0.938 |
| eGFR (mL/min/1.73m^2^) | -0.087 | 0.418 | -0.105 | 0.326 |
| hsCRP (mg/L) | 0.093 | 0.385 | -0.012 | 0.908 |
| FIB-4 index | 0.331 | 0.002 | 0.024 | 0.824 |

Spearman’s rank correlation analysis was performed. All parameters corresponded to the values obtained at baseline. ALT, alanine aminotransferase; AST, aspartate aminotransferase; BMI, body mass index; DBP, diastolic blood pressure; eGFR, estimated glomerular filtration rate; FIB-4, fibrosis 4; GIPR, glucose-dependent insulinotropic polypeptide receptor; GLP-1R, glucagon-like peptide-1 receptor; γ-GTP, γ-glutamyl transpeptidase; HbA1c, hemoglobin A1c; HDL-C, high-density lipoprotein cholesterol; hsCRP, high-sensitivity C-reactive protein; LDL-C, low-density lipoprotein cholesterol; SBP, systolic blood pressure; *ρ,* Spearman's correlation coefficient; TG, triglycerides; UA, uric acid; UACR, urine albumin-to-creatinine ratio.

**Supplementary Table S4. Simple linear regression models examining the association between baseline clinical parameters and percentage change in body weight after 6 months of tirzepatide treatment**

| Dependent variable: Percentage change in body weight | | | |
| --- | --- | --- | --- |
| Independent variables | β coefficient | 95% CI | *P*-value |
| Clinical characteristics |  |  |  |
| Age | 0.157 | 0.113 to 0.202 | <0.001 |
| Female sex | 0.565 | -0.628 to 1.758 | 0.349 |
| Duration of diabetes ^†^ | 1.408 | 0.923 to 1.893 | <0.001 |
| Current smoker | -0.137 | -1.459 to 1.185 | 0.837 |
| Body weight | -0.079 | -0.115 to -0.044 | <0.001 |
| BMI | -0.242 | -0.363 to -0.121 | <0.001 |
| Waist circumference | -0.158 | -0.233 to -0.083 | <0.001 |
| Complications of diabetes |  |  |  |
| Neuropathy | 0.645 | -0.546 to 1.836 | 0.284 |
| Retinopathy | -0.430 | -1.670 to 0.810 | 0.493 |
| Nephropathy | -0.255 | -1.483 to 0.973 | 0.681 |
| Atherosclerotic diseases | 1.219 | -0.151 to 2.589 | 0.081 |
| Physiological testing |  |  |  |
| SBP | -0.017 | -0.079 to 0.045 | 0.587 |
| DBP | -0.062 | -0.161 to 0.037 | 0.217 |
| Laboratory parameters |  |  |  |
| HbA1c ^†^ | 3.888 | -1.155 to 8.931 | 0.129 |
| TG ^†^ | -0.367 | -1.836 to 1.103 | 0.621 |
| HDL-C ^†^ | 3.793 | -0.602 to 8.187 | 0.090 |
| LDL-C ^†^ | 0.294 | -4.279 to 4.866 | 0.899 |
| AST ^†^ | -1.310 | -2.531 to -0.088 | 0.036 |
| ALT ^†^ | -1.975 | -3.421 to -0.529 | 0.008 |
| γ-GTP ^†^ | 0.751 | -0.278 to 1.781 | 0.151 |
| UA ^†^ | -0.037 | -1.872 to 1.798 | 0.968 |
| UACR ^†^ | 0.006 | -0.581 to 0.594 | 0.983 |
| eGFR ^†^ | 2.523 | -1.349 to 6.395 | 0.199 |
| hsCRP ^†^ | -1.077 | -3.163 to 1.009 | 0.308 |
| Data regarding monocytes |  |  |  |
| *GIPR* expression in monocytes ^†^ | -0.960 | -1.272 to -0.648 | <0.001 |
| *GLP-1R* expression in monocytes ^†^ | -0.234 | -0.552 to 0.084 | 0.147 |
| Medications |  |  |  |
| Insulin | -0.280 | -1.777 to 1.217 | 0.711 |
| Sulfonylureas | -0.947 | -2.934 to 1.041 | 0.346 |
| Glinides | -0.772 | -2.872 to 1.327 | 0.467 |
| GLP-1RAs (semaglutide = 1) | 1.659 | 0.514 to 2.805 | 0.005 |
| Metformin | -1.086 | -2.775 to 0.603 | 0.205 |
| TZDs | -1.289 | -3.510 to 0.932 | 0.252 |
| Alpha-GIs | 1.823 | -0.764 to 4.441 | 0.165 |
| SGLT2 inhibitors | -0.323 | -1.545 to 0.898 | 0.600 |
| ARBs | 0.619 | -0.575 to 1.813 | 0.306 |
| CCBs | 0.229 | -1.092 to 1.551 | 0.731 |
| Statins | 0.027 | -1.217 to 1.270 | 0.966 |
| Fibrates | 0.151 | -1.062 to 1.363 | 0.806 |
| UA lowering agents | -0.641 | -2.029 to 0.747 | 0.361 |

All independent variables corresponded to the values obtained at baseline. Percentage change in body weight (%) was calculated as follows: [(body weight at 6 months − baseline body weight) / baseline body weight] × 100. Negative values indicate weight reduction. ALT, alanine aminotransferase; ARBs, angiotensin receptor blockers; AST, aspartate aminotransferase; BMI, body mass index; CCBs, calcium channel blockers; DBP, diastolic blood pressure; eGFR, estimated glomerular filtration rate; γ-GTP, γ-glutamyl transpeptidase; GIPR, glucose-dependent insulinotropic polypeptide receptor; GIs, glycosidase inhibitors; GLP-1R, glucagon-like peptide-1 receptor; GLP-1RAs, glucagon-like peptide-1 receptor agonists; HbA1c, hemoglobin A1c; HDL-C, high-density lipoprotein cholesterol; hsCRP, high-sensitivity C-reactive protein; LDL-C, low-density lipoprotein cholesterol; β, partial regression coefficient; SBP, systolic blood pressure; SGLT2, sodium-glucose cotransporter 2; TG, triglycerides; TZDs, thiazolidinediones; UA, uric acid; UACR, urine albumin-to-creatinine ratio. ^†^ Log-transformed using the natural logarithm.

**Supplementary Table S5. Simple linear regression models examining the association between baseline clinical parameters and change in the FIB-4 index after 6 months of tirzepatide treatment**

| Dependent variable: Change in the FIB-4 index | | | |
| --- | --- | --- | --- |
| Independent variables | β coefficient | 95% CI | *P*-value |
| Clinical characteristics |  |  |  |
| Age | 0.013 | 0.003 to 0.023 | 0.009 |
| Female sex | -0.075 | -0.299 to 0.149 | 0.507 |
| Duration of diabetes ^†^ | 0.196 | 0.098 to 0.294 | <0.001 |
| Current smoker | -0.062 | -0.309 to 0.184 | 0.616 |
| Body weight | -0.005 | -0.012 to 0.002 | 0.165 |
| BMI | -0.018 | -0.042 to 0.006 | 0.148 |
| Waist circumference | -0.008 | -0.024 to 0.007 | 0.273 |
| Complications of diabetes |  |  |  |
| Neuropathy | 0.088 | -0.136 to 0.311 | 0.438 |
| Retinopathy | 0.045 | -0.187 to 0.277 | 0.702 |
| Nephropathy | 0.102 | -0.127 to 0.330 | 0.380 |
| Atherosclerotic diseases | 0.112 | -0.147 to 0.372 | 0.393 |
| Physiological testing |  |  |  |
| SBP | 0.004 | -0.007 to 0.016 | 0.477 |
| DBP | -0.001 | -0.019 to 0.018 | 0.926 |
| Laboratory parameters |  |  |  |
| HbA1c ^†^ | -0.540 | -1.488 to 0.409 | 0.261 |
| TG ^†^ | -0.050 | -0.325 to 0.225 | 0.720 |
| HDL-C ^†^ | 0.577 | -0.249 to 1.403 | 0.168 |
| LDL-C ^†^ | -1.043 | -1.869 to -0.217 | 0.014 |
| AST ^†^ | -0.419 | -0.635 to -0.202 | <0.001 |
| ALT ^†^ | -0.213 | -0.491 to 0.064 | 0.130 |
| γ-GTP ^†^ | 0.036 | -0.159 to 0.230 | 0.717 |
| UA ^†^ | -0.297 | -0.634 to 0.040 | 0.083 |
| UACR ^†^ | 0.058 | -0.051 to 0.167 | 0.293 |
| eGFR ^†^ | -0.112 | -0.842 to 0.619 | 0.762 |
| hsCRP ^†^ | 0.013 | -0.379 to 0.405 | 0.948 |
| Data regarding monocytes |  |  |  |
| *GIPR* expression in monocytes ^†^ | -0.181 | -0.239 to -0.123 | <0.001 |
| *GLP-1R* expression in monocytes ^†^ | -0.030 | -0.090 to 0.029 | 0.315 |
| Medications |  |  |  |
| Insulin | -0.152 | -0.431 to 0.126 | 0.279 |
| Sulfonylureas | -0.259 | -0.629 to 0.110 | 0.167 |
| Glinides | -0.082 | -0.475 to 0.311 | 0.679 |
| GLP-1RAs (semaglutide = 1) | -0.081 | -0.305 to 0.142 | 0.471 |
| Metformin | -0.240 | -0.555 to 0.075 | 0.133 |
| TZDs | -0.240 | -0.655 to 0.175 | 0.254 |
| Alpha-GIs | 0.080 | -0.409 to 0.569 | 0.745 |
| SGLT2 inhibitors | 0.169 | -0.057 to 0.395 | 0.141 |
| ARBs | 0.089 | -0.135 to 0.313 | 0.432 |
| CCBs | 0.015 | -0.233 to 0.262 | 0.907 |
| Statins | -0.057 | -0.289 to 0.175 | 0.626 |
| Fibrates | -0.100 | -0.326 to 0.125 | 0.379 |
| UA lowering agents | -0.054 | -0.315 to 0.206 | 0.679 |

All independent variables corresponded to the values obtained at baseline. The change in FIB-4 index was calculated as the difference between values at 6 months and baseline (FIB-4 index at 6 months − baseline FIB-4 index), with negative values indicating a reduction in the FIB-4 index. ALT, alanine aminotransferase; ARBs, angiotensin receptor blockers; AST, aspartate aminotransferase; BMI, body mass index; CCBs, calcium channel blockers; DBP, diastolic blood pressure; eGFR, estimated glomerular filtration rate; FIB-4, fibrosis 4; γ-GTP, γ-glutamyl transpeptidase; GIPR, glucose-dependent insulinotropic polypeptide receptor; GIs, glycosidase inhibitors; GLP-1R, glucagon-like peptide-1 receptor; GLP-1RAs, glucagon-like peptide-1 receptor agonists; HbA1c, hemoglobin A1c; HDL-C, high-density lipoprotein cholesterol; hsCRP, high-sensitivity C-reactive protein; LDL-C, low-density lipoprotein cholesterol; β, partial regression coefficient; SBP, systolic blood pressure; SGLT2, sodium-glucose cotransporter 2; TG, triglycerides; TZDs, thiazolidinediones; UA, uric acid; UACR, urine albumin-to-creatinine ratio. ^†^ Log-transformed using the natural logarithm.

**Supplementary Table S6. Multiple linear regression analyses examining the association between baseline monocyte GIP receptor mRNA expression and change in the FIB-4 index after 6 months of tirzepatide treatment**

| Dependent variable: Change in the FIB-4 index | | | |
| --- | --- | --- | --- |
|  | β coefficient | 95% CI | *P*-value |
| Age- and sex-adjusted model | (Adjusted *R*^2^ = 0.288) | | |
| *GIPR* expression in monocytes ^†^ | -0.182 | -0.249 to -0.115 | <0.001 |
| Age | 0.0001 | -0.010 to 0.010 | 0.988 |
| Female sex | -0.090 | -0.278 to 0.099 | 0.347 |
| Multiple regression model 1 | (Adjusted *R*^2^ = 0.516) | | |
| *GIPR* expression in monocytes ^†^ | -0.110 | -0.169 to -0.052 | <0.001 |
| Duration of diabetes ^†^ | 0.061 | -0.029 to 0.150 | 0.182 |
| BMI | -0.005 | -0.024 to 0.014 | 0.591 |
| HbA1c ^†^ | -0.001 | -0.711 to 0.710 | 0.999 |
| Baseline FIB-4 index ^†^ | -0.660 | -0.889 to -0.432 | <0.001 |
| Previous GLP-1RA type: semaglutide | -0.120 | -0.286 to 0.047 | 0.158 |
| Multiple regression model 2 | (Adjusted *R*^2^ = 0.516) | | |
| *GIPR* expression in monocytes ^†^ | -0.124 | -0.190 to -0.059 | <0.001 |
| Duration of diabetes ^†^ | 0.074 | -0.020 to 0.167 | 0.122 |
| BMI | -0.007 | -0.026 to 0.012 | 0.460 |
| HbA1c ^†^ | 0.062 | -0.661 to 0.784 | 0.866 |
| Baseline FIB-4 index ^†^ | -0.637 | -0.870 to -0.403 | <0.001 |
| Previous GLP-1RA type: semaglutide | -0.104 | -0.274 to 0.066 | 0.228 |
| Percentage change in body weight | -0.019 | -0.058 to 0.020 | 0.339 |

All independent variables excluding percentage change in body weight corresponded to the values obtained at baseline. The model included covariates selected a priori based on clinical relevance and potential confounding. Baseline FIB-4 index was included instead of its individual components to avoid excessive adjustment and collinearity. Percentage change in body weight (%) was calculated as [(body weight at 6 months − baseline body weight) / baseline body weight] × 100, with negative values indicating weight reduction. The change in FIB-4 index was calculated as the difference between values at 6 months and baseline (FIB-4 index at 6 months − baseline FIB-4 index), with negative values indicating a reduction in the FIB-4 index. BMI, body mass index; CI, confidence interval; FIB-4, fibrosis 4; GIPR, glucose-dependent insulinotropic polypeptide receptor; GLP-1RAs, glucagon-like peptide-1 receptor agonists; HbA1c, hemoglobin A1c; β, partial regression coefficient. ^†^ Log-transformed using the natural logarithm.

**Supplementary Table S7. Sensitivity analysis of the association between baseline monocyte GIP receptor mRNA expression and percentage change in body weight after 6 months of tirzepatide treatment**

| Dependent variable: Percentage change in body weight | | | |
| --- | --- | --- | --- |
|  | β coefficient | 95% CI | *P*-value |
| Crude model | (Adjusted *R*^2^ = 0.238) | | |
| *GIPR* expression in monocytes ^†^ | -0.875 | -1.156 to -0.594 | <0.001 |
| Age- and sex-adjusted model | (Adjusted *R*^2^ = 0.348) | | |
| *GIPR* expression in monocytes ^†^ | -0.590 | -0.878 to -0.302 | <0.001 |
| Age | 0.101 | 0.057 to 0.145 | <0.001 |
| Female sex | 0.443 | -0.362 to 1.247 | 0.278 |
| Multiple regression model | (Adjusted *R*^2^ = 0.388) | | |
| *GIPR* expression in monocytes ^†^ | -0.519 | -0.809 to -0.230 | <0.001 |
| Age | 0.066 | 0.018 to 0.114 | 0.007 |
| Duration of diabetes ^†^ | 0.518 | 0.061 to 0.976 | 0.027 |
| BMI | -0.089 | -0.190 to 0.012 | 0.083 |
| HbA1c ^†^ | 2.954 | -0.498 to 6.407 | 0.093 |
| Previous GLP-1RA type: semaglutide | 0.236 | -0.608 to 1.079 | 0.581 |
| Tirzepatide dose at 6 months: 10 mg/week | -0.445 | -2.273 to 1.383 | 0.631 |
| SGLT2 inhibitor initiation during follow-up | -0.268 | -1.323 to 1.787 | 0.616 |

All independent variables excluding the items related to tirzepatide and SGLT2 inhibitors corresponded to the values obtained at baseline. The sensitivity analysis included 120 participants: 90 participants in the primary analysis and an additional 30 participants who had originally been excluded because of tirzepatide dose escalation to ≥7.5 mg/week (n=6), changes in concomitant medications (n=22), baseline HbA1c >10% (n=1), or severe renal impairment (n=1). Among the six participants who underwent tirzepatide dose escalation, all were receiving 10 mg/week at 6 months. All 22 participants with changes in concomitant medications had newly initiated an SGLT2 inhibitor during the 6-month follow-up period. Percentage change in body weight (%) was calculated as [(body weight at 6 months − baseline body weight) / baseline body weight] × 100, with negative values indicating weight reduction. BMI, body mass index; CI, confidence interval; GIPR, glucose-dependent insulinotropic polypeptide receptor; GLP-1RA, glucagon-like peptide-1 receptor agonist; HbA1c, hemoglobin A1c; SGLT2, sodium-glucose cotransporter 2; β, partial regression coefficient. ^†^ Log-transformed using the natural logarithm.

**Supplementary Table S8. Sensitivity analyses of the association between baseline monocyte GIP receptor mRNA expression and change in the FIB-4 index after 6 months of tirzepatide treatment**

| Dependent variable: Change in the FIB-4 index | | | |
| --- | --- | --- | --- |
|  | β coefficient | 95% CI | *P*-value |
| Crude model | (Adjusted *R*^2^ = 0.156) | | |
| *GIPR* expression in monocytes ^†^ | -0.140 | -0.197 to -0.082 | <0.001 |
| Age- and sex-adjusted model | (Adjusted *R*^2^ = 0.151) | | |
| *GIPR* expression in monocytes ^†^ | -0.124 | -0.188 to -0.060 | <0.001 |
| Age | 0.006 | -0.004 to 0.015 | 0.261 |
| Female sex | -0.003 | -0.182 to 0.176 | 0.975 |
| Multiple regression model 1 | (Adjusted *R*^2^ = 0.445) | | |
| *GIPR* expression in monocytes ^†^ | -0.082 | -0.133 to -0.030 | 0.002 |
| Duration of diabetes ^†^ | 0.039 | -0.043 to 0.120 | 0.348 |
| BMI | -0.007 | -0.026 to 0.012 | 0.450 |
| HbA1c ^†^ | -0.081 | -0.734 to 0.573 | 0.807 |
| Baseline FIB-4 index ^†^ | -0.779 | -0.985 to -0.573 | <0.001 |
| Previous GLP-1RA type: semaglutide | -0.040 | -0.197 to 0.117 | 0.618 |
| Tirzepatide dose at 6 months: 10 mg/week | -0.266 | -0.604 to 0.072 | 0.122 |
| SGLT2 inhibitor initiation during follow-up | -0.003 | -0.199 to 0.193 | 0.977 |
| Multiple regression model 2 | (Adjusted *R*^2^ = 0.444) | | |
| *GIPR* expression in monocytes ^†^ | -0.092 | -0.149 to -0.035 | 0.002 |
| Duration of diabetes ^†^ | 0.050 | -0.035 to 0.136 | 0.246 |
| BMI | -0.009 | -0.028 to 0.010 | 0.364 |
| HbA1c ^†^ | -0.034 | -0.696 to 0.628 | 0.918 |
| Baseline FIB-4 index ^†^ | -0.766 | -0.974 to -0.558 | <0.001 |
| Previous GLP-1RA type: semaglutide | -0.035 | -0.192 to 0.123 | 0.664 |
| Tirzepatide dose at 6 months: 10 mg/week | -0.276 | -0.615 to 0.063 | 0.110 |
| SGLT2 inhibitor initiation during follow-up | -0.007 | -0.203 to 0.190 | 0.947 |
| Percentage change in body weight | -0.015 | -0.049 to 0.019 | 0.378 |

All independent variables excluding percentage change in body weight and the items related to tirzepatide and SGLT2 inhibitors corresponded to the values obtained at baseline. The sensitivity analysis included 120 participants: 90 participants in the primary analysis and an additional 30 participants who had originally been excluded because of tirzepatide dose escalation to ≥7.5 mg/week (n=6), changes in concomitant medications (n=22), baseline HbA1c >10% (n=1), or severe renal impairment (n=1). Among the six participants who underwent tirzepatide dose escalation, all were receiving 10 mg/week at 6 months. All 22 participants with changes in concomitant medications had newly initiated an SGLT2 inhibitor during the 6-month follow-up period. Baseline FIB-4 index was included instead of its individual components to avoid excessive adjustment and collinearity. Percentage change in body weight (%) was calculated as [(body weight at 6 months − baseline body weight) / baseline body weight] × 100, with negative values indicating weight reduction. Change in the FIB-4 index was calculated as the difference between values at 6 months and baseline (FIB-4 index at 6 months − baseline FIB-4 index), with negative values indicating a reduction in the FIB-4 index. BMI, body mass index; CI, confidence interval; FIB-4, fibrosis 4; GIPR, glucose-dependent insulinotropic polypeptide receptor; GLP-1RA, glucagon-like peptide-1 receptor agonist; HbA1c, hemoglobin A1c; SGLT2, sodium-glucose cotransporter 2; β, partial regression coefficient. ^†^ Log-transformed using the natural logarithm.

**Supplementary Table S9. Comparison of baseline clinical characteristics between participants included in the primary analysis and patients without analyzable baseline monocyte samples**

|  | Primary analysis cohort (*n* = 90) | Patients without analyzable baseline monocyte samples (*n* = 43) | *P-*value |
| --- | --- | --- | --- |
| Clinical characteristics |  |  |  |
| Age (years) | 57.5±10.8 | 59.4±10.9 | 0.343 |
| Female sex, *n* (%) | 44 (49) | 20 (47) | 0.854 |
| Duration of diabetes (years) | 11 (3-21) | 13 (3-22) | 0.893 |
| Current smoker (*n*, %) | 26 (29) | 16 (37) | 0.425 |
| Body weight (kg) | 74.9±15.4 | 78.1±12.2 | 0.235 |
| BMI (kg/m^2^) | 27.6±4.6 | 28.5±3.6 | 0.310 |
| Waist circumference (cm) | 89.7±7.3 | 90.7±7.8 | 0.454 |
| Complications of diabetes (*n*, %) |  |  |  |
| Neuropathy | 45 (50) | 19 (44) | 0.581 |
| Retinopathy | 33 (37) | 22 (51) | 0.134 |
| Nephropathy | 55 (61) | 30 (56) | 0.440 |
| Atherosclerotic diseases | 22 (24) | 8 (30) | 0.512 |
| Physiological testing |  |  |  |
| SBP (mmHg) | 135±10 | 133±9 | 0.563 |
| DBP (mmHg) | 71±6 | 72±7 | 0.384 |
| Laboratory parameters |  |  |  |
| HbA1c (%) | 8.05 (7.50–9.10) | 8.20 (7.50–9.30) | 0.520 |
| TG (mg/dL) | 246 (186–327) | 260 (186–337) | 0.744 |
| HDL-C (mg/dL) | 44 (40–49) | 43 (39–48) | 0.656 |
| LDL-C (mg/dL) | 110 (95–120) | 109 (95–119) | 0.795 |
| AST (IU/L) | 30 (19–36) | 30 (25–37) | 0.469 |
| ALT (IU/L) | 39 (29–47) | 44 (32–49) | 0.166 |
| γ-GTP (IU/L) | 54 (34–71) | 67 (35–79) | 0.236 |
| UA (mg/dL) | 5.9 (4.3–7.1) | 6.2 (5.3–7.7) | 0.110 |
| UACR (mg/gCr) | 39.9 (17.2–76.6) | 55.6 (18.2–80.8) | 0.407 |
| eGFR (mL/min/1.73m^2^) | 70.8 (60.4–80.0) | 71.3 (62.4–79.2) | 0.769 |
| hsCRP (mg/L) | 2.9 (2.5–3.6) | 3.2 (2.7–3.8) | 0.257 |
| FIB-4 index | 1.18 (0.94–1.56) | 1.25 (0.95–1.75) | 0.419 |
| Medications (*n*, %) |  |  |  |
| Insulin | 18 (20) | 12 (28) | 0.376 |
| Sulfonylureas | 9 (10) | 7 (16) | 0.393 |
| Glinides | 8 (9) | 7 (16) | 0.245 |
| GLP-1RAs (semaglutide) | 45 (50) | 25 (58) | 0.459 |
| Metformin | 77 (86) | 39 (91) | 0.580 |
| TZDs | 7 (8) | 4 (9) | 0.746 |
| Alpha-GIs | 5 (6) | 4 (9) | 0.470 |
| SGLT2 inhibitors | 54 (60) | 27 (63) | 0.850 |
| ARBs | 42 (47) | 25 (58) | 0.267 |
| CCBs | 26 (29) | 16 (37) | 0.425 |
| Statins | 57 (63) | 30 (70) | 0.560 |
| Fibrates | 38 (42) | 20 (47) | 0.710 |
| UA lowering agents | 22 (24) | 15 (35) | 0.221 |

Data are expressed as the mean ± standard deviation, median (interquartile range), or number (%). ALT, alanine aminotransferase; ARBs, angiotensin receptor blockers; AST, aspartate aminotransferase; BMI, body mass index; CCBs, calcium channel blockers; DBP, diastolic blood pressure; eGFR, estimated glomerular filtration rate; FIB-4, fibrosis 4; γ-GTP, γ-glutamyl transpeptidase; GIs, glycosidase inhibitors; GLP-1RAs, glucagon-like peptide-1 receptor agonists; HbA1c, hemoglobin A1c; HDL-C, high-density lipoprotein cholesterol; hsCRP, high-sensitivity C-reactive protein; LDL-C, low-density lipoprotein cholesterol; SBP, systolic blood pressure; SGLT2, sodium-glucose cotransporter 2; TG, triglycerides; TZDs, thiazolidinediones; UA, uric acid; UACR, urine albumin-to-creatinine ratio.

**SUPPLEMENTARY FIGURE**

**Supplementary Fig. S1. Association between baseline monocyte GIP receptor mRNA expression and body-weight change during the 6 months preceding tirzepatide initiation**

The association between monocyte *GIPR* mRNA expression measured at tirzepatide initiation and percentage change in body weight during the preceding 6 months of GLP-1RA treatment was evaluated in all 90 participants using Spearman’s rank correlation analysis. Percentage change in body weight was calculated as [(body weight at tirzepatide initiation − body weight 6 months before tirzepatide initiation) / body weight 6 months before tirzepatide initiation] × 100. ACTB, actin beta; GIPR, glucose-dependent insulinotropic polypeptide receptor; GLP-1RA, glucagon-like peptide-1 receptor agonist; ρ, Spearman’s rank correlation coefficient.

**Supplementary Fig. S2. Exploratory subset analysis of GIP-induced cAMP responses in peripheral blood monocytes**

Intracellular cAMP levels were measured in peripheral blood monocytes from 23 participants with sufficient residual cell samples. Cultured monocytes were stimulated with vehicle or 1 nM human GIP (1–42) for 30 min. (a) Comparison of intracellular cAMP levels between vehicle- and GIP-stimulated monocytes. Data were analyzed using the Wilcoxon signed-rank test. All data are presented as box-and-whisker plots. The box represents the interquartile range, the horizontal line within the box indicates the median, and the whiskers represent the 10th and 90th percentiles. The median values are indicated at the bottom of a panel. (b) The correlation between baseline monocyte *GIPR* mRNA expression levels and the GIP-induced cAMP response was evaluated using Spearman’s rank correlation analysis. The GIP-induced cAMP response was calculated as the cAMP level after GIP stimulation divided by the cAMP level after vehicle stimulation. ACTB, actin beta; cAMP, cyclic adenosine monophosphate; GIP, glucose-dependent insulinotropic polypeptide; GIPR, glucose-dependent insulinotropic polypeptide receptor; ρ, Spearman’s correlation coefficient. ***P < 0.001.
